# Mobile Application for Tracking Children with Autistic Spectrum Disorder: Content Validation and Usability

**DOI:** 10.3390/ijerph21121590

**Published:** 2024-11-29

**Authors:** Camila Porpino Maia Garcia, Andrea Gondim Mendonça, Adriane da Cunha Aragão Rios Fagundes, Ana Cristina de Macedo Santos, Francilene Jane Rodrigues Pereira, Stella Costa Valdevino, Rebecca Maria Oliveira de Góis, Rodrigo Barros Esteves Lins, Renata Pascoal Freire, Mailson Marques de Sousa, Vagna Cristina Leite da Silva Pereira, Cleyton Cézar Souto Silva

**Affiliations:** 1 Nova Esperança Nursing School, João Pessoa 58067-698, PB, Brazil; camilamaiagarcia@gmail.com (C.P.M.G.);; 2 Federal University of Rio Grande do Norte, Natal 59078-900, RN, Brazil; 3 Lauro Wanderley University Hospital, João Pessoa 58050-585, PB, Brazil; 4 Department of Clinical Nurse, Federal University of Paraíba, João Pessoa 58050-585, PB, Brazilmailson.sousa@academico.ufpb.br (M.M.d.S.); 5 School of Dentistry, Federal University of Alagoas, Maceió Alagoas 57072-900, AL, Brazil; rodrigo.lins@foufal.ufal.br; 6 Federal University of Santa Catarina, Florianópolis 88040-900, SC, Brazil; renatapfreire@gmail.com

**Keywords:** autism spectrum disorder, validation study, evaluation study, mobile applications, growth and development

## Abstract

Background: This study aims to validate the content and evaluate the usability of the Autism App for screening children with autism spectrum disorder. Methods: This methodological study was conducted between August 2023 and March 2024. The study included 15 experts for content validation and nine for usability evaluation, all of whom had experience in the health/technology field. Content validation data were analyzed using the Content Validity Ratio (CVR), and usability was assessed through heuristics using the MATcH-MED instrument. Results: The content validation showed satisfactory CVR values, ensuring the validity of the developed application, with suggestions for revisions regarding aims and structure. The usability evaluation yielded an overall average score of 50.9 points, classified as a high level of usability. Conclusion: The results demonstrated that the App-Autismo has adequate evidence of content validity and usability. By incorporating the experts’ suggestions, this tool can be tested in future research for its effectiveness and efficiency.

## 1. Introduction

Autism spectrum disorder (ASD) is described as a neurodevelopmental impairment that leads to deficits in communication and social interaction, as well as a pattern of restricted and repetitive behaviors, interests and activities, which typically manifest before three years of age and may sometimes be associated with intellectual disability [1].

A recent study has shown that despite the increased awareness of early signs of autism, late identification remains a global issue [2]. A systematic review revealed that diagnoses occur at an average age of 5 years, with significant variability between the earliest and latest age of diagnosis [3].

Another integrative review study indicates that the use of early diagnostic instruments is still incipient, with the implementation of these evaluative questionnaires in primary healthcare being a significant challenge [4]. Given the importance of early diagnosis and intervention, the American Academy of Pediatrics (AAP) recommends that all children be screened in primary healthcare between 18 and 24 months of age [5].

Early diagnosis enhances and amplifies the possibilities for intervention during the early stages of child development by enabling the development of cognitive skills, such as verbal language and communication; socio-cognitive skills, such as shared attention; and behavioral skills, such as autonomy and social abilities. It also improves guidance for parents and caregivers through the development of management strategies [6].

Increasing evidence has shown advancements in the use of mobile health (m-Health) applications designed to meet the complex needs of children with ASD and neurodevelopmental disorders, suggesting potential efficacy in enhancing healthcare, particularly in terms of accessibility for the population [7].

One example is a mobile application developed and validated with a high index for teaching about nursing appointments for newborns and their families in primary care, which includes content on neonatal screening, growth and development [8].

A scoping review identified a greater number of tools with scientific evidence for monitoring the growth and development of infants in mobile applications [9]. Another investigation found that the Modified Checklist for Autism in Toddlers (M-CHAT) is the most accurate instrument for early screening and monitoring of autism symptoms, demonstrating high sensitivity and reliability [10].

The availability of a mobile app that facilitates early screening for ASD can enable early interventions and lead to better developmental outcomes for children with ASD, including improvements in cognition, language, daily routine activities and social behavior [11].

Currently, the monitoring of growth and development (GD) has shown deficiencies, both in the correct entry of data in the Child Health Booklet and in the efficient performance of GD surveillance. In fact, many consultations are limited to monitoring anthropometric data, which can lead to vulnerabilities in children’s health that are further intensified by a lack of skill and technical knowledge when managing disorders that interfere with neurodevelopment [12].

Thus, there is a need to help health professionals expand their knowledge in order to facilitate the practice of child surveillance through adequate monitoring and the identification of possible neurodevelopmental delays at an early stage [13].

The App-Autismo was developed to contribute to the early identification of warning signs for ASD in an accessible and intuitive way, facilitating the use of the M-CHAT-R scale in the monitoring of child growth and development by primary healthcare (PHC) health professionals who carry out consultations known as Childcare in Health Units [9].

The need to develop the app arose as a result of a scoping study carried out with the aim of mapping health technologies for monitoring the growth and development of infants, highlighting the need for apps aimed at health professionals with an educational and care nature [9]. Another review study found that the M-CHAT-R is the most accurate instrument for early screening and monitoring of symptoms of the disorder, with high sensitivity and reliability, and was therefore included in the structure of the Autism App [10].

The effective use of a mobile application depends on building an appropriate project and meeting user expectations, since poorly designed software can lead to dissatisfaction, increased error rates and decreased safety and quality of patient care services [14].

App-Autismo was developed using the analysis, design, development, implementation and evaluation (ADDIE) model, which is divided into small interdependent phases, in which planning (analysis, design, development) is separated from the execution of the app (implementation and evaluation) [9,10]. According to this model, the development stages of the mobile application followed the following stages:

- Stage 1—Analysis: This consisted of delimiting the educational problem and designing an approximate solution, through a situational analysis with a survey of needs and information, which was consolidated through a scoping review that mapped the technologies available on the subject and the literature review carried out on ASD screening in PHC.
- Stage 2—Design: This involved planning the design of the situation itself, by mapping and sequencing the content, structured by the work of graphic design professionals who helped produce the application’s visuals, focused on the educational and assistance objectives.
- Stage 3—Development of the app prototype: This consisted of producing and adapting teaching resources and materials, parameterizing virtual environments and preparing teaching, technological and administrative support. This phase included the presentation of content on ASD to be included in the app, the definition of the target audience for the content and the smartphone platforms.

The health technology assessment stage is described as an interdisciplinary process, involving professionals from various fields, such as medicine, engineering, economics, statistics, mathematics and research. These specialists apply defined methods to analyze the value of health technologies and provide suggestions on their use. The process of evaluating and validating technological products must be carried out in a systematic and transparent way and can count on evaluating judges and the participation of the target public to help with health decisions, presenting evidence on new interventions and knowledge [15].

Thus, in order to develop an application in a coherent and appropriate manner, it is essential to identify the specific needs of the target audience, so that the application can be designed to fulfill its functionality, validated in research and implemented in practice [16].

Therefore, the process of developing an app involves different methodological stages, and it is essential to assess the quality of the idealized technology. This judgment can be made through content validation and usability evaluation research [13]. The validation and evaluation process verifies that the product does exactly what it sets out to do [17].

Given the above, the following question arises: Is the App-Autismo a valid mobile device in terms of content and usability for screening children with ASD in primary healthcare? The aim of this study was to validate the content and evaluate the usability of the App-Autismo by expert judges.

## 2. Materials and Methods

### 2.1. Study Type

This is a methodological study focusing on content validation and heuristic evaluation of the usability of the App-Autismo prototype. The App-Autismo was developed on 45 screens, intended for both assistive and educational purposes. It has a simple, attractive and user-friendly interface, with theoretical content organized into “themes,” allowing for practical and secure individualized access to information about ASD [13].

The app was built using the ADDIE model for online use on iOS and Android platforms [10]. Its development took place in stages, described below:

Planning: The availability of technologies on the market was assessed, as were the development methodologies used, the most relevant programming language for development and the main platforms for analyzing the number of users, access facilities and post-development use.

Requirements analysis: A survey was carried out of the functional, non-functional and regulatory requirements of the application that the system should contain. Functional requirements included the implementation of text boxes with information about ASD. Non-functional requirements included ease and speed of use, as well as broad access to and understanding of the solution developed.

Coding: After gathering the definitions for the content through a literature review [10], the data collected was presented in a suitable format so that it could later be coded in computer language and embedded in the software, in a robust and integrated development environment, in accordance with the requirements defined in the planning phase. The object-oriented programming language paradigm was used, with the dynamism, portability, high performance and security of the Java language.

Development: The way in which the application would be used and the level of quality of use were considered throughout its development, looking at its functions, interfaces, and who will interact with them and handle the system. The environment in which the application would be used was taken into account, and a series of points were observed throughout the construction of the application.

The App-Autismo software was developed with the contribution of a product designer through a contracted service for the artistic elaboration of the work, thus assigning the copyright to the author as an individual, so she is the owner and is responsible for the work in the literary and scientific sphere [18].

The content defined on the basis of literature reviews was developed in the app in relation to the concept of ASD, its etiology, risk factors, diagnostic signs and symptoms, assessment of growth and development in childcare consultations, as well as interactive content such as the child’s booklet, a video on the surveillance of child growth and development, lines of care on ASD and the M-CHAT-R questionnaire directly in the app, which provided the scoring parameters for assessing the risk of ASD.

The validation stage of educational and care technologies is considered to be of fundamental importance and a complex analysis that requires appropriate pedagogical approaches and methods. If the validation stage is not carried out, the material produced can be considered inadequate and non-functional [19].

Content validation refers to expert judgment and the app’s ability to cover all important aspects of the problem being investigated. In this study, content validation of the mobile application was carried out using the Content Validity Ratio (CVR), which aims to obtain agreement among expert judges, thereby reducing the risk of bias related to the size of the expert panel, as the critical value of the CVR depends on the number of experts included [20]. At this stage, a minimum of five judges is recommended [21].

Among the validation methods, CVR stands out as a sophisticated method compared to other proposed techniques, as it takes into account the number of expert judges and minimizes the inflation of random agreement. It is also considered an excellent psychometric indicator, capable of providing the best assessment of content validity, which aims to obtain agreement between expert judges [20].

In order to promote better usability of the App-Autismo, the basic principles followed during the development of the software were considered: minimum user effort; more recognition of functions than demands on the user’s memory; minimum frustration during handling; increased use based on work patterns and habits; tolerance for differences between the people who will use the system; changes in the possible environments in which the system will be used; presence of communication interfaces for notifying problems; maximum support for these tasks by the system [14].

Usability is defined as a quality attribute that assesses how easy interfaces are for users. By evaluating usability levels, it is possible to assess the software’s efficiency (speed of task execution), ease of use, design, learnability, memorability, error rates and user satisfaction in interacting with an application [22].

Among the expert-based inspection methods, the heuristic evaluation method is an evaluation methodology that can recognize up to 80% of system usability problems in a practical, fast and economical way [14,23].

Thus, the choice of the “Checklist for Evaluating the Usability of m-Health Applications on Smartphones (MATcH-MED)”, over others, was due to the fact that the instrument was developed to evaluate usability exclusively for m-Health applications on smartphones based on Nielson’s 12 heuristics [24].

Nielsen’s heuristics for mobile devices were used through the checklist defining a set of 12 usability heuristics in the health area: visibility and system status; correspondence between application and real world; user control and freedom; consistency and standards; error prevention; recognition rather than recall; flexibility and efficiency of use; aesthetics and minimalist design; minimized human-computer interaction; physical interaction and ergonomics; readability and quick visualization; workflow [25].

### 2.2. Study Population

The study population consisted of a panel of experts in the field of health/technology. To be considered eligible for content validation and usability evaluation, the experts had to meet criteria adapted from Fehring [26]. According to Fehring, the minimum score is five and the maximum is fourteen points; judges scoring below the minimum are considered to have insufficient knowledge of the subject and are therefore excluded from the evaluation [26].

The selection of experts for content and usability evaluation was conducted through non-probabilistic and intentional sampling, specifically using the snowball method, which facilitates and broadens the selection of judges with similar profiles who meet the study’s interest [27]. Research was conducted on the Lattes Curriculum platform of the National Council for Scientific and Technological Development (CNPq) and in articles published in journals on the topic of mobile app development and validation, where the authors met the desired profile for selection. The expert panel consisted of 15 judges for content validation and 9 judges for usability evaluation. Based on the recommendations of the Brazilian Association of Technical Standards (ABNT) for software product evaluation, eight or more people are recommended for usability testing [18,22].

Potential experts were invited via email. Upon acceptance, they received a Google Forms weblink via email with the instruments for content validation or usability evaluation and a weblink to download the mobile application to their mobile device, with a 30-day deadline for submission. The links for accessing this system were as follows:

- <https://play.google.com/store/apps/details?id=com.ELO.ELO>(Android);
- <https://eloapp.vercel.app/>(iOS).

### 2.3. Instruments and Data Analysis

The instrument for content validation of the app was structured in two parts: (1) identification of academic and professional data—age, gender, area of expertise, time since graduation, position/role in the current institution, length of professional experience and academic qualifications; (2) organization into three blocks: I—objectives and content, II—structure and functionality, and III—relevance, adapted from Teixeira and Mota [28].

In this study, the cut-off points for the Likert scale were adjusted in 3 blocks, with the CVR (Content Validity Ratio) calculated for each variable in the instrument, presenting the following valuation options: essential: a combination of 1 (totally adequate) and 2 (adequate); useful but not essential: 3 (partially adequate); and unnecessary: 4 (inadequate).

The CVR assesses the relevance of content based on the agreement among experts regarding how much a particular item can be considered “essential to the test,” “useful to the test but not essential,” or “not necessary” [29,30]. According to the formula CVR = Ne − (N/2)/N/2, where Ne is the number of expert judges who marked the score “essential,” and N is the number of respondents. For a panel of 15 content experts, a critical CVR of 0.60 was considered. Values below this cut-off point were considered for re-evaluation and correction of suggestions [30].

Usability evaluation was conducted using the MATcH-MED Checklist, which consists of 43 items across 12 heuristics and is available at http://match.inf.ufsc.br:90/matchmed/ (accessed on 1 January 2023). According to the MATcH-MED analysis, the application in question can fall into one of five levels: very low usability (up to 30); low usability (30–40); reasonable usability (40–50); high usability (50–60); and very high usability (above 60) [25].

### 2.4. App-Autismo Content and Resources

The app allows users to log in by entering their e-mail address and password. From this screen, you can access the “About the app” link, which takes you to information about the app’s objective and purpose.

The “introduction” button describes the definition and prevalence of ASD, etiology, risk factors and the line of care published by the Ministry of Health. The “diagnosis” option presents the warning signs, the environment for diagnosing ASD, a link to the Child’s Handbook, complementary exams and comorbidities.

The option “assessing child development” emphasizes the importance of monitoring developmental milestones in hearing, vision and aspects of the motor system. The “assessing risk” button mentions the legislation that makes it compulsory to apply a screening tool to children for developmental alterations, presenting the M-CHAT-R scale and its correlation of answers with scores.

The “putting it into practice” button allows you to apply the M-CHAT-R scale, which can be viewed widely by scrolling. After completing items 1 to 20, the total score will be displayed and the health behavior will be available at the bottom of the screen. Finally, the “final message” button emphasizes the importance of using the tool as a screening tool for early diagnosis and intervention. It also suggests using the child’s report card as an important tool for approaching child health.

### 2.5. Ethical Aspects

The study was submitted to the Research Ethics Committee (IRB) and approved under CAAE number 69180123.0.0000.5179 and opinion number 6.141.301. The participants formalized their consent by signing the Informed Consent Form (TCLE).

## 3. Results

The content expert panel consisted of fifteen evaluators from the healthcare field, including fourteen women (93.33%) and one man (6.67%). In terms of their area of expertise, eleven (73.33%) were nurses, two (13.33%) were physiotherapists, one (6.67%) was a psychologist, and one (6.67%) did not report their professional category. Ten (66.67%) held a PhD, and five (33.33%) held a Master’s degree. The average age was 38.93 (±9.60) years, ranging from 21 to 64 years. Additionally, the average lengths of education and professional experience were 17.07 (±8.32) years and 13.12 (±9.00) years, respectively.

Table 1 presents the results of the instrument’s content validation. The CVR for items A2 and B5 did not meet the minimum score of 0.60, with scores of 0.47 and 0.20, respectively, regarding the guidance to parents on the importance of pediatric care and the size and type of font.

Regarding the profiles of the nine experts who evaluated usability, five (55.55%) were women and four (44.44%) were men. In terms of their area of expertise, eight (88.88%) were nurses with experience in the development and evaluation of applications, and one (11.11%) had a background in computing. According to their qualifications, eight (88.88%) held PhDs and one (11.11%) was a specialist. The average age of the usability experts was 41.44 (±9.80) years, ranging from 32 to 64 years. Additionally, the average length of education and professional experience was 17.67 (±10.84) years and 10.83 (±11.91) years, respectively.

Table 2 presents the average scores obtained from the heuristic evaluation of the mobile application by usability experts for each item of the MATcH-MED.

The overall mean usability score, obtained by summing the MATcH-MED scores, was 50.9, classified as high usability. The highest observed usability score was 61.7, and the lowest was 38.5 (Table 3).

The suggested modifications by the experts are presented below in Table 4.

**Figure 1:**
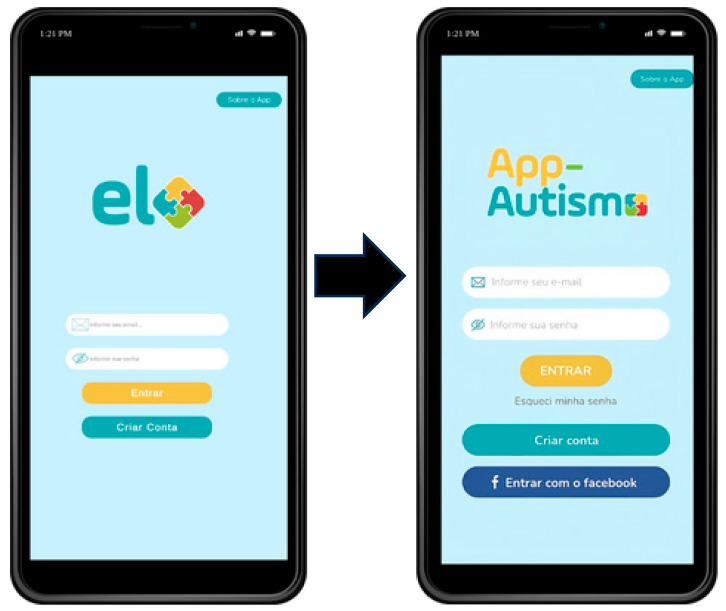
Login screen (before and after the application name change).

**Figure 2:**
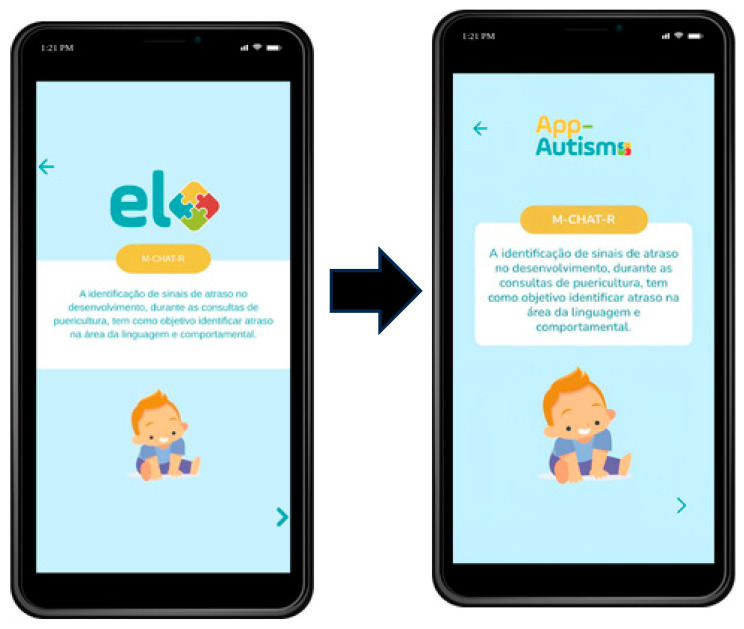
M-CHAT-R initial screen (before and after font size modification).

The experts considered that the content presented has appropriate language and could be used as a reference in the care pathway, along with the Brazilian Ministry of Health’s Child Health Booklet. The main changes made to the screens of the application, which is now titled App-Autismo, are shown in the following Figure 1 and Figure 2.

**Figure 3:**
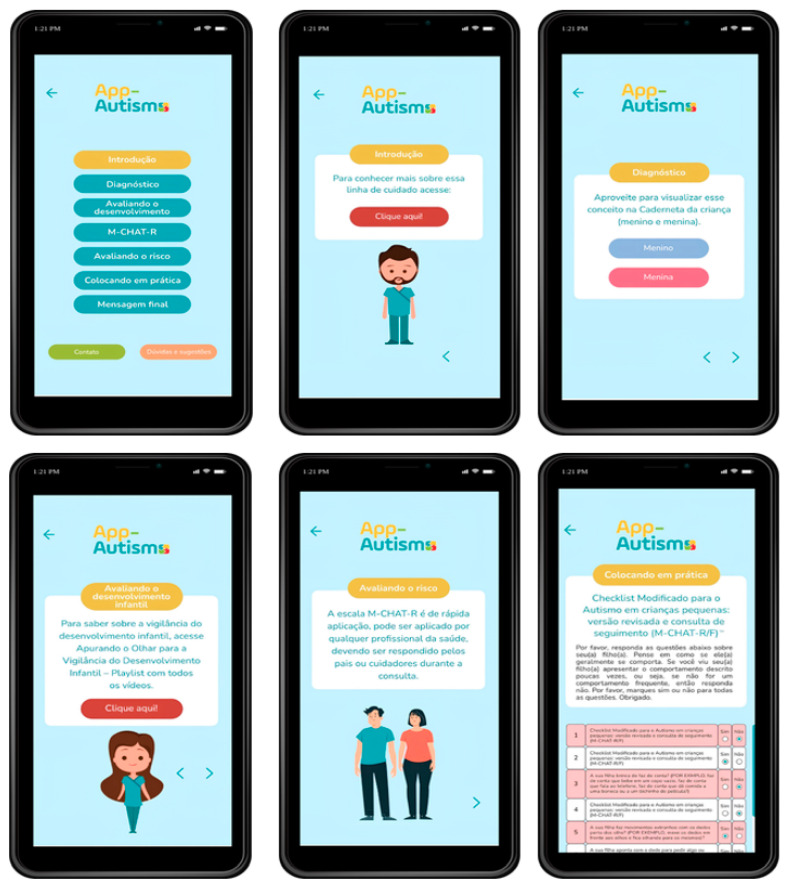
Some screens of the App-Autismo.

The App-Autismo is scientifically grounded, covering content related to ASD, including signs and symptoms, etiology, risk factors, diagnosis and risk assessment, aimed at primary healthcare professionals (Figure 3).

## 4. Discussion

The purpose of the App-Autismo is to help PHC health professionals in screening children with ASD, presenting adequate evidence as to its content and usability, and thus being able to be used as a technological tool to support the Child Health Booklet during growth and development consultations in PHC.

Early identification of developmental alterations and immediate intervention with early stimulation, followed by timely referral and appropriate diagnosis, results in a better therapeutic prognosis. To support health professionals in this practice, the App-Autismo features an expansive menu with multiple-choice buttons that lead to topics such as: introduction, diagnosis, assessing development, M-CHAT-R and its recommendation for application at the 18-month appointment, assessing risk, putting it into practice and final message. The Child Health Booklet, available in a “boy” and “girl” version, is also available with different colored buttons (blue and pink, respectively) [13].

Regarding content validation, concerning the objective of guiding parents on the importance of pediatric care, it was found that this item had a CVR below the established cut-off point. It is important to note that the purpose of this app is not to provide direct guidance to parents and caregivers so that they can use it independently. Instead, its aim is to be associated with the growth and development consultations of health professionals in PHC units, with the active participation of these guardians. In any case, some of the experts’ suggestions were accepted, especially with regard to the need for clear language for using the App-Autismo, both in a care and educational way, during childcare. The aim is to promote the participation and responsibility of parents and caregivers in relation to children diagnosed with a risk of ASD.

It is noteworthy that the App-Autismo includes screens with guidance texts on contributing factors to the development of ASD, clinical diagnosis with the main signs and symptoms, online access to the line of care available in the Health Network of the Unified Health System (SUS), as well as the Child Health Booklet, with girl and boy versions, authored by the Ministry of Health. Thus, the mobile app provides reliable information for healthcare professionals to guide parents and family members on caring for a child with ASD.

Similarly, the Breastfeeding Coach app consists of numerous screens with texts, videos, infographics and other resources on breastfeeding, lactation and prematurity for mothers with babies in intensive care units. After corrections suggested by 10 evaluators, the mobile application achieved a coefficient of content validation and appearance, with the potential to positively impact the breastfeeding experience of mothers with premature babies [31].

Regarding structure, the CVR for the item mentioning text formatting concerning font type and size also had a value below the recommended threshold. Experts suggested increasing the font size and type to facilitate reading and accommodate all audiences. Additionally, it was proposed to change the app’s name to App-Autismo due to its similarity to the trademarks of other commercial brands. Other CVR values were satisfactory, ensuring the validity of the developed app and demonstrating its appropriateness. Some modification suggestions were accepted to improve the app, such as changing the font size and type for clearer screen visibility.

In contrast to the validation of App-Autismo, the developers of the Baby Date app did not accept the suggested modifications to alternate text placement, images and font size, arguing that such changes would hinder quick access to information and affect the intent to highlight specific content for nursing appointments and health education with parents and caregivers of newborns [8]. The decision to change the font size and font of the App-Autismo does not directly affect future development and/or expansion interactions, as its new source code will be updated according to the improvements requested.

Currently, some apps have been developed to meet the needs of children and newborns; however, they still have limitations, such as being available only for research purposes and not for download from app stores, while others lack adequate validity evidence [11].

The App-Autismo introduces as innovation the possibility of detecting ASD early in children and referring them to healthcare specialists as soon as possible, allowing for stimulation and monitoring throughout their development. One of its features is the M-CHAT-R scale, a validated instrument for the early detection of ASD that is guaranteed by the Brazilian Ministry of Health as a care and diagnostic tool [32,33].

It is emphasized that the App-Autismo is not intended to replace face-to-face clinical care by health professionals, but rather to enhance the screening of children at risk of ASD in a practical way, through interactive resources with access to videos and links to the Child Health Booklet.

Studies have shown that the development of mobile devices in healthcare, particularly during the validation stage, contributes to the production of shared and qualified knowledge, expanding the discussion of specific points in each field of action based on improvement suggestions [26,34].

Regarding usability evaluation, the App-Autismo achieved an average score of 50.9, classified as high usability. At this level of stratification, it is considered that the information is presented logically and naturally, images and other interface components are concrete and familiar, data entry and navigation are minimal, visual design is attractive and “clean,” buttons are appropriately spaced, visual cues are used to separate unrelated content, and information is easily viewable [25].

These results reflect the quality of the App-Autismo, with no usability issues identified. Similarly, another study used the MATcH-MED to evaluate the usability of a gamified electromyographic biofeedback mobile app and achieved a high average score of 56.82 points, making it suitable for monitoring voluntary contraction of mastication and swallowing muscles [35].

The App-Autismo represents an innovative clinical and educational tool, enabling the dissemination of knowledge about ASD, identification of risk factors for its development, personal data storage, access to care pathways, interactivity with the Child Health Booklet, monitoring of child development and the M-CHAT for screening health risks. Therefore, health professionals can learn more about possible signs of ASD by filling in a scale made up of “yes” or “no” questions together with parents and guardians. The questions were developed based on a list of frequent symptoms in children with autism [36].

One limitation of the study is the participation of professionals in the mobile app validation with representation from only some regions of the country, rather than its entirety. Future research is needed to assess the effectiveness of the mobile app’s applicability among healthcare professionals conducting child growth and development appointments through intervention studies.

## 5. Conclusions

The App-Autismo for screening children with ASD has adequate evidence of content validity and usability. The CVR showed satisfactory values, guaranteeing the validity of the content and demonstrating the app’s suitability for use by PHC health professionals in order to guarantee comprehensive care and quality care for children with autism spectrum disorder. Usability obtained an average score of 50.9, which classifies it as having a high level of usability. This data is crucial for the app’s credibility and can serve as a reference for future developments of similar technologies in the health area.

The app has great potential to improve public health outcomes by enabling the early identification of signs of ASD, which is essential for agile interventions and for promoting better development in children. In addition, it offers accessibility by making information and interactive resources available to health professionals in PHC, helping to overcome obstacles related to the treatment of ASD.

We hope to incorporate the technology to support the clinical and educational practice of PHC professionals with parents and guardians as an important tool for reaching a diagnosis. Its application should be associated with the use of the Child Health Booklet in childcare consultations, guaranteeing appropriate interventions in the Health Care Network for the cases identified. This may inspire other researchers to explore how applications can be incorporated into health systems to improve healthcare.

In addition, the App-Autismo adds value to the existing literature in several ways, as it represents an innovative application that uses M-CHAT for early screening of ASD, contributing to the literature by offering a new tool that can be used by healthcare professionals, improving early detection and referral to appropriate interventions. The app not only helps with screening but also serves as an educational resource, contributing to the ongoing training of professionals and the education of parents and caregivers.

Finally, by focusing on a significant public health problem, the article highlights the social relevance of App-Autismo. The emphasis on early screening for ASD can positively impact the lives of many children and their families, which is an important aspect to consider in the literature on public health and technology.

## Figures and Tables

**Table 1:** Evaluated topics regarding content. João Pessoa, PB, Brazil, 2024.

Aspects	Items	I-CVR
Aims	A1—The content is appropriate for the target audience.	0.73
	A2—The app provides guidance to parents on the importance of child healthcare (puericulture).	0.47
	A3—The app clarifies doubts about signs of ASD	0.60
	A4—The displayed content is scientific.	0.73
Structure	B1—The app provides useful tools for monitoring children with ASD.	0.87
	B2—The messages are displayed clearly and objectively.	0.60
	B3—The distribution of information follows an appropriate order	0.73
	B4—The text has good agreement and spelling.	0.87
	B5—The font size and type make the content easy to read.	0.20
	B6—The illustrations (images and GIFs) are clear.	0.73
	B7—The number of screens is satisfactory.	0.87
	B8—The app interface is attractive.	0.87
Relevance	C1—The app addresses evident topics related to ASD.	1.00
	C2—The app features items for monitoring child growth and development.	0.60
	C3—The app provides content for identifying ASD.	0.60
	C4—The app can be used by healthcare professionals during health education.	0.73

Source: Research data from 2024. I-CVR: Item Content Validity Ratio.

**Table 2:** Average scores of usability experts for each MATcH-MED item. João Pessoa, PB, Brazil, 2024.

Heuristic	Items	Mean (SD)	Min	Max
Visibility of the app’s status	1. The user is informed about what is happening in the app through feedback and the display of information in a clear, concise, and appropriate manner.	2.56 (1.13)	1	4
	2. Selected components are highlighted, while disabled components are “grayed out” or omitted.	2.00 (1.22)	1	4
	3. Critical and contextual information, such as battery status, date/time, internet connection, etc., is prioritized and visible.	2.11 (1.27)	1	4
Match between the app and the real world.	4. Control labels are consistent with their actions.	1.78 (1.30)	1	4
	5. Information appears in a logical and natural order.	1.00 (0.00)	1	1
	6. Menu options and labels can be quickly understood.	1.33 (1.00)	1	4
	7. Icons, images, and other interface components are concrete and familiar.	2.00 (1.22)	1	4
	8. The colors used align with common color-coding expectations.	1.67 (1.12)	1	4
	9. The language used is always understandable to users.	1.56 (1.13)	1	4
User control and freedom	10. Users can navigate forward and backward in the app.	1.67 (1.32)	1	4
	11. Users can save tasks in progress to continue later.	1.89 (0.93)	1	3
Consistency and standards	12. Interface elements follow standard terminology.	1.44 (0.88)	1	3
	13. App navigation and screen layout are consistent.	1.33 (1.00)	1	4
	14. The interface style is consistent across the app’s screens.	1.33 (1.00)	1	4
	15. The app follows platform conventions.	1.22 (1.00)	1	3
Error prevention	16. Valid ranges for parameters (minimum and maximum limits) are indicated.	2.78 (0.83)	1	4
	17. Menu options are logical, distinct, and mutually exclusive.	1.00 (0.00)	1	1
	18. The app requires complex procedures to confirm risky actions that might cause accidental errors.	2.33 (1.00)	1	4
Recognition rather than recall	19. All information necessary for users to complete tasks is visible and/or easy to find.	1.89 (1.17)	1	4
	20. A unique color code is used for quick identification and recall.	1.89 (0.93)	1	3
	21. The app provides all necessary information.	1.89 (1.36)	1	4
	22. Menus are balanced, neither too deep nor too wide.	1.33 (1.00)	1	4
Efficiency and flexibility	23. Key functionalities of the app are easy to access.	1.00 (0.00)	1	1
	24. Related functionalities are close to each other.	1.33 (0.71)	1	3
	25. The time required to complete a task is appropriate.	1.00 (0.00)	1	1
	26. Data entry and navigation are minimal.	1.00 (0.00)	1	1
	27. The need to use scrolling is avoided.	1.44 (1.01)	1	4
Aesthetic and minimalist design	28. The visual design is attractive.	1.67 (1.32)	1	4
	29. Screens have a “clean” design, showing only important information and components.	1.00 (0.00)	1	1
	30. Screen content is always fully visible and not covered by other interface components.	1.44 (1.01)	1	4
	31. The available screen space is maximized.	1.33 (1.00)	1	4
Physical interaction and ergonomics	32. Touch action components are appropriately sized for users to easily tap with their fingers +47. The touch area of controls is the same size as the icon displayed on the screen.	1.67 (1.32)	1	4
	33. Buttons are spaced adequately to prevent users from pressing the wrong one.	1.33 (1.00)	1	4
Readability and quick visualization	34. Information can be viewed quickly.	1.33 (1.00)	1	4
	35. Important information is highlighted.	2.00 (1.12)	1	4
	36. There is good color and brightness contrast between images, text, icons, and background.	2.00 (1.50)	1	4
	37. Visual cues are used to separate unrelated content.	1.22 (0.67)	1	3
	38. Content is easy to read.	1.67 (1.32)	1	4
	39. Text is presented in an organized manner.	1.33 (1.00)	1	4
	40. Icons and images have appropriate size and resolution.	1.78 (1.30)	1	4
	41. Text fields fit on the screen.	1.33 (1.00)	1	4
Workflow	42. It is clear where to start activities.	1.00 (0.00)	1	1
	43. The flow of screens matches the flow of user activities.	1.33 (1.00)	1	4

Source: Research data from 2024.

**Table 3:** Distribution of scores and usability level of experts. João Pessoa, PB, Brazil, 2024.

Judges-Specialists	MATcH-MED Total Score	Usability Level
1	52.3	HIGH
2	44.7	MODERATE
3	54	HIGH
4	61.7	VERY HIGH
5	57.4	HIGH
6	45.6	MODERATE
7	38.5	BAIXA
8	60.6	VERY HIGH
9	44	MODERATE
Average	50.9	HIGH

Source: Research data from 2024.

**Table 4:** Suggestions for improving the app from the experts in terms of content. João Pessoa, PB, Brazil, 2024.

Aspects	Comments	Changes
Aims	The app does not provide guidance to parents on child healthcare (puericulture).	The mobile app was not developed for parents; however, it was indirectly emphasized that healthcare professionals should use it during child healthcare appointments.
Structure	Some screens have font size and type that are too small for proper visibility and readability on the screen.	The font size was adjusted on all screens where it was previously inadequate.
Structure	Change the app’s name to avoid duplication with a commercial brand of the same name.	A new name design was created, while maintaining the previous colors and symbols.
Relevance	There were no suggestions for improvements.	-

## Data Availability

The data records of the experts used for this study are not available due to restrictions required by the ethics committee on research involving human beings. A reasonable set of anonymized metadata and derived data used for the cluster analysis can be obtained from the corresponding author.
